# Clock gene *Per1* regulates rat temporomandibular osteoarthritis through NF-κB pathway: an in vitro and in vivo study

**DOI:** 10.1186/s13018-023-04301-7

**Published:** 2023-10-31

**Authors:** Jia-ming Wei, Shao-qin Tu, Yu-xuan Wang, Sai Zhang, Yi Feng, Hong Ai, Zheng Chen

**Affiliations:** Department of Stomatology, The Third Affiliated Hospital of Sun Yat-sen University, Guangdong, China

**Keywords:** Circadian rhythm, *Per1*, NF-κB, TMJOA, Chondrocytes

## Abstract

**Purpose:**

Temporomandibular joint osteoarthritis (TMJOA) is a common disease that negatively affects the life quality of human beings. Circadian rhythm acts an important role in life activities. However, whether the clock genes are rhythmic expressed in mandibular condylar chondrocytes, or the clock genes have an effect on the progression of TMJOA remains unknown. In this study, we aim to explore expression of clock genes and regulatory mechanism of TMJOA in rat mandibular condylar chondrocytes.

**Methods:**

After synchronized by dexamethasone, the expression of core clock genes *Per1*, *Per2*, *Clock*, *Cry1*, *Cry2* and *Bmal1* and cartilage matrix degrading factor gene *Mmp13* were analyzed in mandibular condylar chondrocytes every 4 h with RT-qPCR. The mandibular condylar chondrocytes were stimulated with IL-1β, and expression of *Per1*, *Mmp13*, P65 and p-P65 was assessed by RT-qPCR and Western blot. Sh-*Per1* lentivirus was used to assess the effect of clock gene *Per1* in IL-1β-induced chondrocytes, and expression of *Mmp13*, P65 and p-P65 was measured. After establishing a rat TMJOA model using unilateral anterior crossbite (UAC), micro-CT, H & E, Alcian Blue & Nuclear Fast Red and Safranin O & Fast Green, cartilage thickness was utilized to assess the damage of cartilage and subchondral bone. Immunohistochemistry of PER1, MMP13 and P65 was performed in condylar sections.

**Results:**

All core clock genes and *Mmp13* were rhythmically expressed. And *Mmp13* expression curve was closed in phase and amplitude with *Per1*. After stimulation with IL-1β, the expression of MMP13, PER1 and P65 and ratio of p-P65/P65 increased in condylar chondrocytes. After *Per1* was down-regulated in condylar chondrocytes, the expression of MMP13 and P65 and ratio of p-P65/P65 decreased. Compared with the condyles of Sham group, the bony parameters of UAC group were significantly worse. The thickness of cartilage in UAC group significantly reduced. The modified Mankin scores and the expression of PER1, MMP13 and P65 in cartilage of UAC group significantly increased compared with Sham group.

**Conclusion:**

Core clock genes and *Mmp13* are rhythmic expressed in rat mandibular condylar chondrocytes. PER1 can regulate the expression of MMP13 through NF-κB pathway in IL-1β-induced mandibular condylar chondrocytes.

**Supplementary Information:**

The online version contains supplementary material available at 10.1186/s13018-023-04301-7.

## Introduction

Temporomandibular joint osteoarthrosis (TMJOA) is a common manifestation of temporomandibular joint disorder that often presents with bone resorption, condylar cysts or osteophyte formation [1, 2]. Severe cases can lead to joint dyskinesia, mouth restriction, etc. Condylar resorption can even lead to mandibular retraction and other facial deformities, seriously affecting the living quality and psychological health of patients [3]. Its etiology includes local factors such as injury, parafunction, occlusion and joint overload [4], as well as systemic factors such as age, sex, autoimmune diseases and hormonal disorders [5]. Mandibular condylar chondrocytes are the only cells in condylar cartilage, so the factors affecting the proliferation, differentiation and mineralization of condylar chondrocytes will affect the growth and reconstruction of condyles, which can eventually be manifested as condylar resorption [6–8]. Early cartilage degeneration is caused by metabolic or local mechanical factors, which trigger an immune response. And then, immune cells release cytokines such as IL-1β, TNF-α, etc., and other inflammatory factors to initiate the inflammatory response, which increases the synthesis of cartilage matrix degrading factors, including matrix metalloproteinases (MMPs) and prostaglandin E (PGE). These factors further aggravate the decomposition of cartilage and eventually lead to the degeneration of articular cartilage, which, in turn, leads to the resorption and degeneration of subchondral bone [2, 9, 10].

Circadian rhythm is a continuously biological rhythm regulated by the clock genes, with a cycle of about 24 h. It is a periodic oscillation of biochemical activities in the organism, regulating human body temperature, hormone, sleep, blood pressure and other life activities [11, 12]. There is also a circadian clock in the peripheral tissues, which is regulated by the central clock. After the central clock is destroyed, the peripheral clock will gradually disappear [13]. The circadian rhythm is regulated by multiple clock genes. At present, more than ten core clock genes have been found, including *Bma1*, *Clock*, *Cry1*, *Cry2*, *Per1*, *Per2*, *Rev-erb α*, *ROR α*, etc. *Bmal1* and *Clock* are positive regulatory clock genes, and *Cry1*, *Cry2*, *Per1*, *Per2*, *Rev-erb α* and *ROR* α are negative regulatory genes [14, 15]. There are rhythmic changes in cartilage metabolism, such as the proliferation of chondrocytes mainly occurring in the early morning and mineralization mainly occurring at night. The expression of various matrix proteins in cartilage, such as hyaluronic acid (HA) and collagen II, also is rhythmic expressed [16–18]. Studies have shown that compared with the knee cartilage of normal individuals, the expression of *NR1D1* and *BMAL1* in the knee cartilage of osteoarthritis (OA) patients is relatively low, and the expression of *Bmal1* is also lower in the OA rat model, which is negatively correlated with the severity of OA [19, 20]. In the OA mice model, it was found that the expression of clock gene *Per2* reduced [21]. The clock gene *Cry1* may be involved in anti-COL2 antibody-induced mouse OA model and exacerbate the progression of osteoarthritis [22].

There are few studies on whether there is a peripheral clock in the mandibular condylar chondrocytes, and whether the clock gene is involved in the progression of TMJOA. This study is aimed to explore whether clock genes are rhythmic expressed in condylar chondrocytes in vitro, investigate whether clock genes are abnormally expressed through TMJOA model in vitro and vivo and preliminarily explore possible mechanism.

## Materials and methods

### Isolation and culture of mandibular condylar chondrocytes

The temporomandibular joint cartilage was isolated from 15 male Sprague Dawley rats (3–5 d) in the present study. After digested with 0.25% trypsin and 0.1 collagenase type I for 2 h at 37 °C, the chondrocytes were collected by centrifugation at 1000 rpm/min and cultured with DMEM (Gibco, USA) containing 20% fetal bovine serum (FBS, Gibco, USA), 100-IU penicillin and 100-μg/mL streptomycin (Gibco, USA) in incubator at 37 °C with 5% CO_2_. Cells in passage 2 were used for further experiments.

### Circadian rhyme synchronization of mandibular condylar chondrocytes

After passaging the cells to passage 2, the chondrocytes were treated with a known synchronizer of peripheral clock (100-nM dexamethasone) for 1 h [21, 23]. And then, the total RNA was extracted from cultured chondrocytes using TRIzol (Invitrogen, USA) every 4 h and reverse transcribed to cDNA using PrimeScript™ RT Master Mix (Takara, Japan). All the cDNA samples were subsequently conducted to polymerase chain reaction with Hieff UNICON® Universal Blue qPCR SYBR Green Master Mix (Yeasen, China). The expression of clock genes (*Per1, Per2, Clock, Cry1, Cry2 and Bmal1*) and *Mmp13* was calculated by using cycle threshold method (2^−△△Ct^). The primer sequence was attached in Additional file 1. And the internal control gene was *Gapdh*. All experiments were repeated three times. The relative expression of genes at a given time was normalized as a percent value of mean level across all the time points. The results were fitted with trigonometric functions by Origin Pro 10.0 (OriginLab, USA).

### Cell treatment and gene inhibition

After synchronized with 100-nM dexamethasone for 1 h, the mandibular chondrocytes were treated with 10-ng/mL IL-1β for 24 h [24, 25] to simulate temporomandibular joint osteoarthritis in vitro. Total RNA and protein were extracted from IL-1β-treated cells. In order to inhibit the expression of *Per1*, the chondrocytes were transfected with sh-*Per1* and sh-control vectors via lentiviruses (Genechem, China) for 24 h. And the cells were stimulated with IL-1β following inhibition of *Per1* by lentiviruses for next 24 h. After that, total RNA and protein were extracted at the same time point during a day. Western blot and RT-qPCR were performed to detect the expression of *Mmp13* and *Per1*.

### Western blot

The collected protein samples were quantified using BCA method (Yeasen, China) and separated by 10% SDS-PAGE. Then, the protein was transferred onto the PVDF membrane (Millipore, Germany). After blocking with 5% non-fat milk at room temperature for 1 h, the membrane was incubated with primary antibodies including rabbit anti-PER1 (Affinity, USA, 1:800), rabbit anti-MMP13 (Proteintech, China, 1:1000), rabbit anti-NF-kB p65 (Affinity, USA, 1:500), rabbit anti-Phospho-NF-kB p65 (Ser536) (Affinity, USA, 1:500) and rabbit anti-GAPDH (Affinity, USA, 1:2000) at 4 °C for 16 h. Following the washing with 0.1% TBST, the secondary antibody (Beyotime, China) was incubated with the membrane. After washing with 0.1% TBST, a chemiluminescence ECL system (Millipore, Germany) was used to visualize the immunoreactive proteins.

### Unilateral anterior crossbite (UAC)-induced TMJOA

All operations are carried out in accordance with animal ethics (approved by Ethics Committee of Sun Yat-sen University, China). Fourteen male Sprague Dawley rats (6 w, 150–160 g) were randomly divided into experimental group and control group, with seven rats in each group. Prior to the start of the experiment, the rats were acclimated for 1 week under a 12-h light/12-h dark environment (8:00 AM–8:00 PM light and 8:00 PM–8:00 AM dark), and the conditions for the formal experiment are consistent with the conditions stated above. The animals were housed with two in a cage in a room maintained at 21–24 °C. All animals were fed a regular laboratory diet with daily changing soft feed and sterile water. After intraperitoneal injection of 1% pentobarbital sodium (0.3 mL/100-g body weight) as anesthesia, we used 25# cow lacteal needle to make the upper anterior tooth restoration and 20# needle to make the lower anterior tooth restoration. The lower restoration made the angle of 135° with the upper anterior tooth restoration, forming unilateral anterior crossbite according to Wang’s method [26]. All the rats ate soft feed after surgery, periodically check whether the restoration has fallen off and bond in time if it falls off. After 8 weeks, the rats were sacrificed, and the condyles were isolated at the same time point during a day for subsequent experiments.

### Micro-CT

Condyles of rats in each groups were collected and fixed with 4% paraformaldehyde solution for 24 h and then scanned by micro-CT (70 kV, 114 mA, 6.8 μm, Scanco.μCT 50). The parameters including bone surface/bone volume (BS/BV), bone volume fraction (BV/TV), trabecular thickness (Tb.Th), trabecular separation (Tb.Sp) and trabecular number (Tb.N) were analyzed. Three-dimensional images were reconstructed by μCT evaluation Program V6.6 for morphological assessment.

### Histological analysis

Condyles of rats were demineralized in 10% ethylenediaminetetraacetic acid for 8 weeks and then embedded with paraffin after dehydration. About 5-μm mid-sagittal sections parallel to condyle were cut and dewaxed in xylene, hydrated in gradient alcohol. H & E, Alcian Blue & Nuclear Fast Red and Safranin O & Fast Green staining were applied according to protocol. Modified Mankin score and cartilage thickness [27, 28] were calculated according to the histological sections to assess severity of TMJOA.

### Immunohistochemistry

Dewaxing and hydration were performed as we described previously. We used 3% hydrogen peroxide to block endogenous peroxidase activity for 15 min. And the sections were antigen-retrieved in citrate solution by microwave. Following by serum blocking for 1 h and primary antibody including rabbit anti-PER1 (Affinity, USA, 1:100), rabbit anti-MMP13 (Proteintech, China, 1:300) and rabbit anti-NF-κB p65 (Affinity, USA, 1:100) at 4 °C were incubated overnight individually. Secondary antibody was incubated for 1 h after PBS washing. Finally, we used DAB method to visualize the immunoreactive cells and hematoxylin to stain the nucleus. We used ImageJ 1.52i (National Institutes of Health, Germany) to measure the average optical density in anterior, middle and posterior fields of each section.

### Statistical analysis

Data were performed by Prism 8.0 (GraphPad, USA). Student’s *t*-test (two-tailed) was used to compare means of two groups, and one-way ANOVA was used to compare means of three or more groups. Test level was *α* = 0.05. All the quantitative data were presented as mean ± SEM in figures.

## Results

### Expression of core clock genes and *Mmp13* is rhythmic

**Fig. 1:**
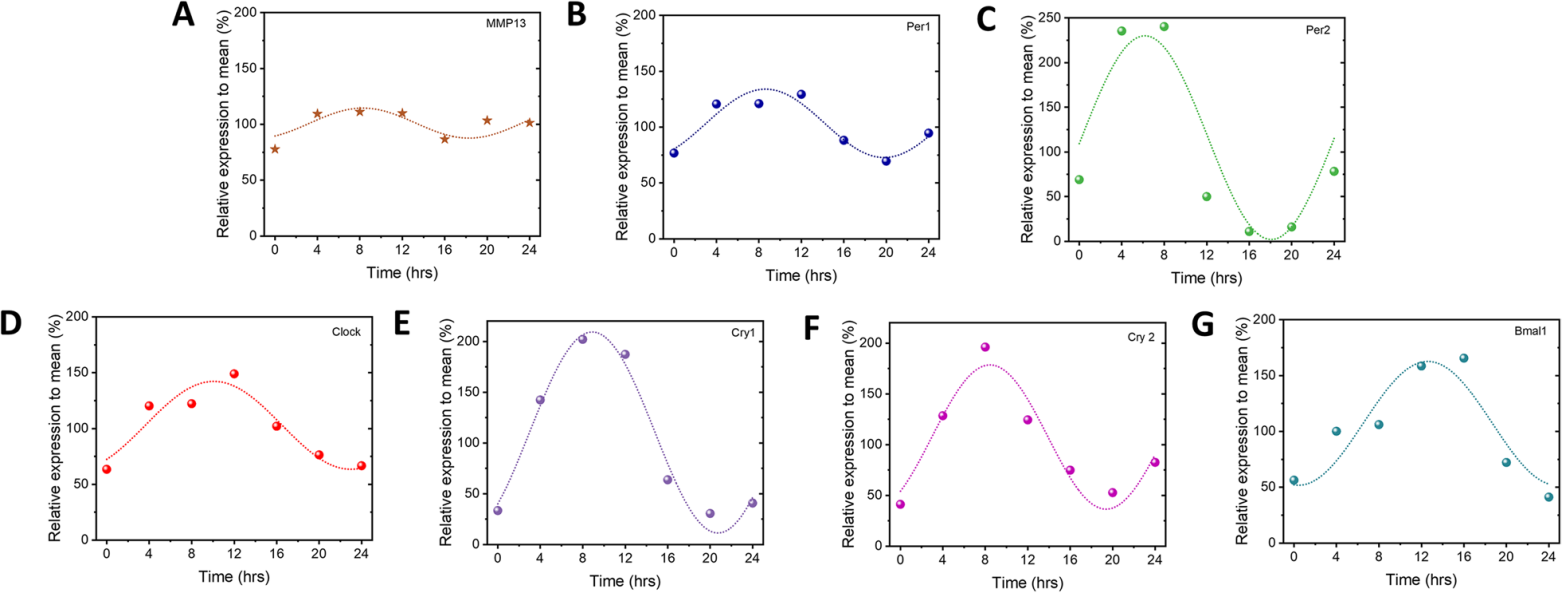
*Mmp13* and core clock genes are rhythmically expressed in rat mandibular condylar chondrocytes after synchronization of dexamethasone. **A** Expression of *Mmp13* within 24 h.** B** Expression of *Per1* within 24 h. **C** Expression of *Per2* within 24 h. **D** Expression of *Clock* within 24 h. **E** Expression of *Cry1* within 24 h. **F** Expression of *Cry2* within 24 h. **G** Expression of *Bmal1* within 24 h

In order to explore the existence of peripheral clock in mandibular condylar chondrocytes, we used the dexamethasone to synchronize the circadian rhythm and measured the mRNA expression of core clock genes and *Mmp13*. All core clock genes and *Mmp13* (Fig. 1) were rhythmically expressed. We calculated the functions of all the genes using Origin software and found that the function of *Per1* (Fig. 1B) is closest to the function of *Mmp13* (Fig. 1A) in terms of phase and amplitude, which indicates a possible relationship between clock gene *Per1* and *Mmp13*. Therefore, we chose the clock gene *Per1* as the target gene for further study.

### IL-1β increases the expression of *Per1* and *Mmp13* and activates NF-κB pathway in mandibular condylar chondrocytes

**Fig. 2:**
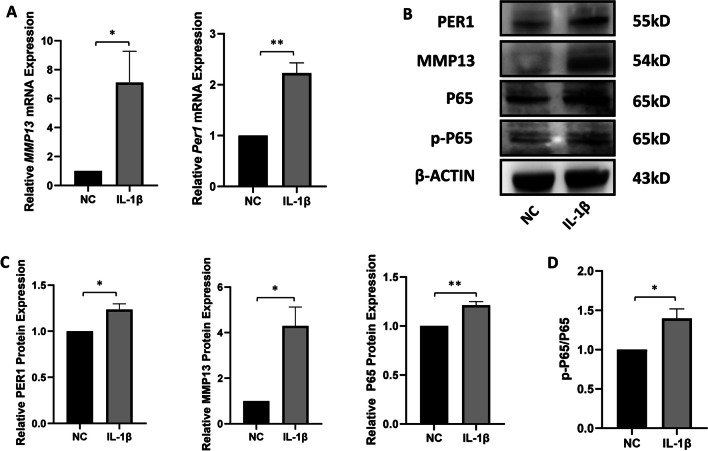
IL-1β increases the expression of *Per1* and *Mmp13* and activates the NF-κB pathway in mandibular condylar chondrocytes**. A** The mRNA levels of *Mmp13* and *Per1* after stimulation of IL-1β in chondrocytes. **B**, **C** Protein level and analysis of MMP13, PER1, P65 and p-P65 after stimulation of IL-1β in chondrocytes. **D** The ratio of p-P65/P65 in two groups. Values are displayed as the mean ± SEM. *P < 0.05 and **P < 0.01

To simulate TMJOA in vitro, we added IL-1β to the rat mandibular chondrocytes. After stimulation with IL-1β, the mRNA and protein expression levels of *Mmp13* and *Per1* and protein expression level of P65 and p-P65 were measured using RT-qPCR and Western blot. As shown in Fig. 2A–C, mRNA and protein levels of *Mmp13* and *Per1* in chondrocytes significantly increased compared to control group. It indicates that clock gene *Per1* may have a potential positive correlation with *Mmp13*. And we examined the NF-κB pathway-related proteins in the IL-1β-induced chondrocytes. As shown in Fig. 2C and D, the protein expression level of P65 (*P* < 0.01) and ratio of p-P65/P65 (*P* < 0.05) significantly increased after stimulation of IL-1β.

### Down-regulation of *Per1* inhibits the expression of *Mmp13* and NF-κB pathway induced by IL-1β

**Fig. 3:**
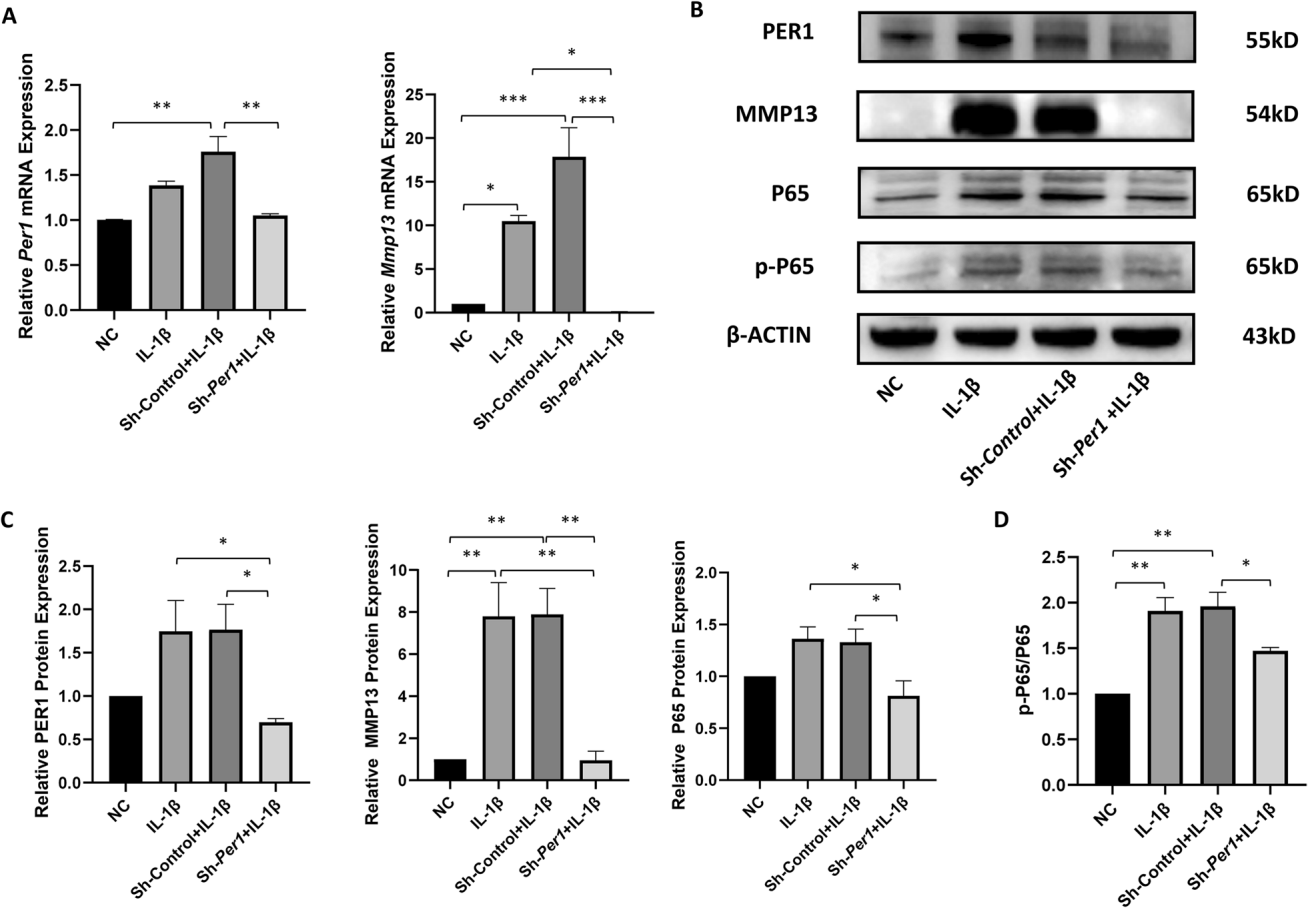
Down-regulation of *Per1* inhibits the expression of *Mmp13* and NF-κB pathway induced by IL-1β.** A** RT-qPCR analysis of *Per1* and *Mmp13* in chondrocytes of NC, IL-1β, sh-control+IL-1β and sh-*Per1*+IL-1β groups. **B** Western blot analysis of PER1, MMP13, P65 and p-P65 in four groups. **C** The relative protein expression level of PER1, MMP13, P65 and p-P65 in four groups. **D** The ratio of p-P65/P65 in four groups. Values are displayed as the mean ± SEM. *P < 0.05, **P < 0.01 and ***P < 0.001

To further explore the correlation between clock gene *Per1* and *Mmp13*, and potential regulatory mechanism, we examined the expression of *Mmp13* and NF-κB pathway-related proteins with *Per1* down-regulation. After down-regulation of *Per1* by lentiviruses, the mRNA and protein levels of *Per1* in IL-1β-induced mandibular condylar chondrocytes were significantly reduced compared with sh-control+IL-1β group (Fig. 3A-B, mRNA level *P* < 0.01. Protein level *P* < 0.05). The efficacy of sh-*Per1* was verified. Compared with the sh-control group, sh-*Per1* lentivirus significantly reduced the mRNA (*P* < 0.001) and protein (*P* < 0.01) levels of *Mmp13*. And down-regulation of *Per1* also significantly inhibited the expression of P65 (Fig. 3B-C) and ratio of p-P65/P65 (Fig. 3D). Take together, these results indicate that clock gene *Per1* can activate the expression of *Mmp13* through NF-κB pathway in IL-1β-induced mandibular condylar chondrocytes.

### Degenerative lesions were observed in unilateral anterior crossbite TMJOA rat model

**Fig. 4:**
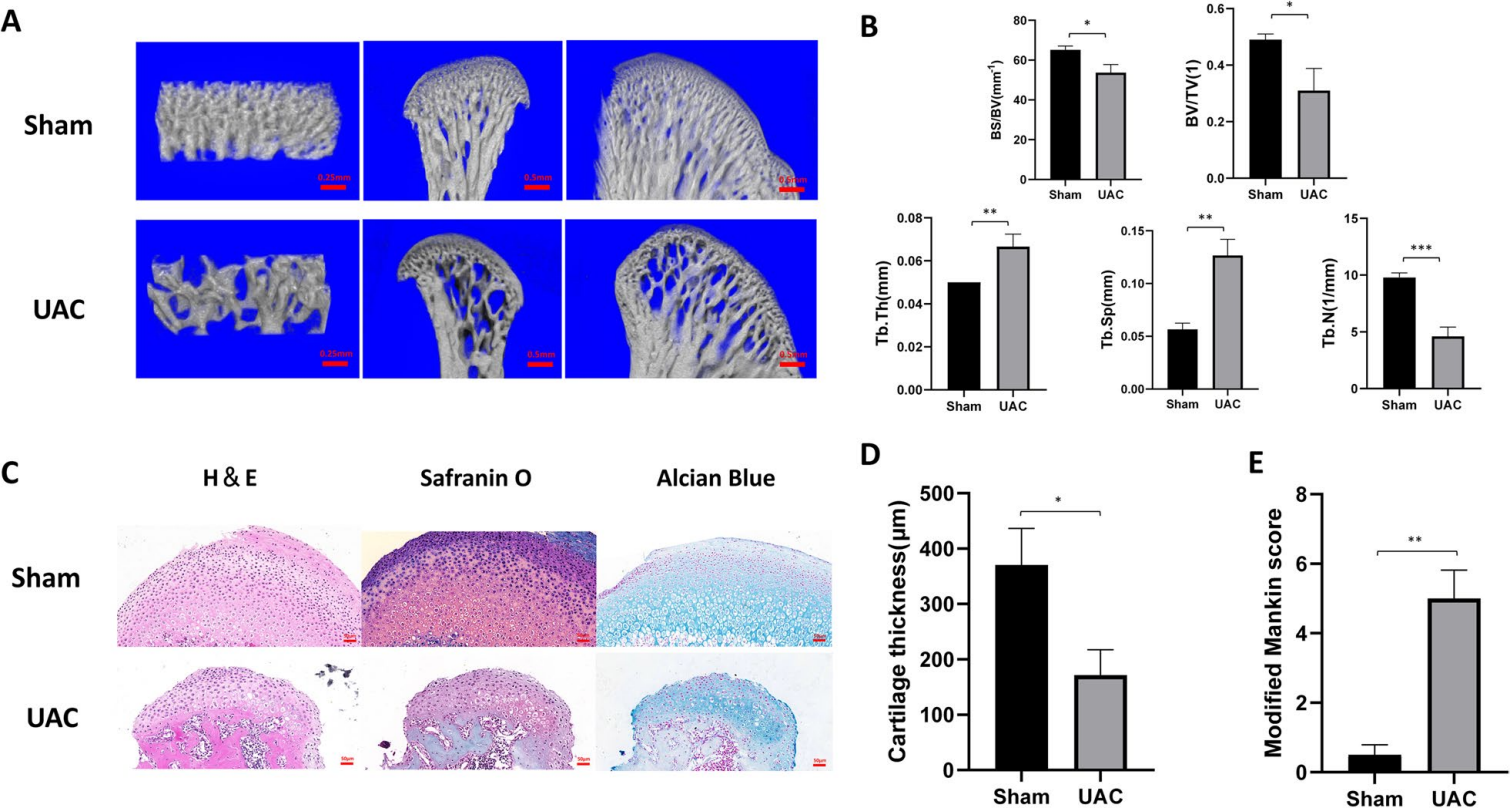
Degenerative lesions are observed in unilateral anterior crossbite TMJOA rat model. **A** Three-dimensional reconstruction of condyles in UAC group and Sham group. **B** Quantitative evaluation including BS/BV, BV/TV, Tb.Th, Tb.Sp and Tb.N of two groups (*n* = 3). **C** H & E, Alcian Blue & Nuclear Fast Red and Safranin O & Fast Green staining of two groups (*n* = 4). **D** The thickness of cartilage in two groups (*n* = 4). **E** The modified Mankin scores of two groups (*n* = 4). Values are displayed as the mean ± SEM. *P < 0.05, **P < 0.01 and ***P < 0.001

To explore the relationship between PER1 and MMP13 in vivo, we established a TMJOA rat model through unilateral anterior crossbite. As shown in micro-CT analysis, there were obvious bone lesions in 3D reconstruction images of UAC group. Both the thickness of the cartilage layer and the density of the subchondral trabeculae are significantly reduced in UAC group compared with Sham group (Fig. 4A). Similarly, quantitative evaluation results (Fig. 4B) including BS/BV, BV/TV, Tb.SP and Tb.N in UAC group significantly decreased compared with Sham group. The Tb.Th in UAC group was significantly higher than the Sham group. As Fig. 4D shown, the thickness of cartilage was significantly thinner (*P* < 0.05) in UAC group than Sham group. The modified Mankin score (Fig. 4E) in UAC group was also significantly higher than the Sham group (*P* < 0.01).

### The expression of PER1, MMP13 and P65 increased in the mandibular condylar cartilage of UAC rats

**Fig. 5:**
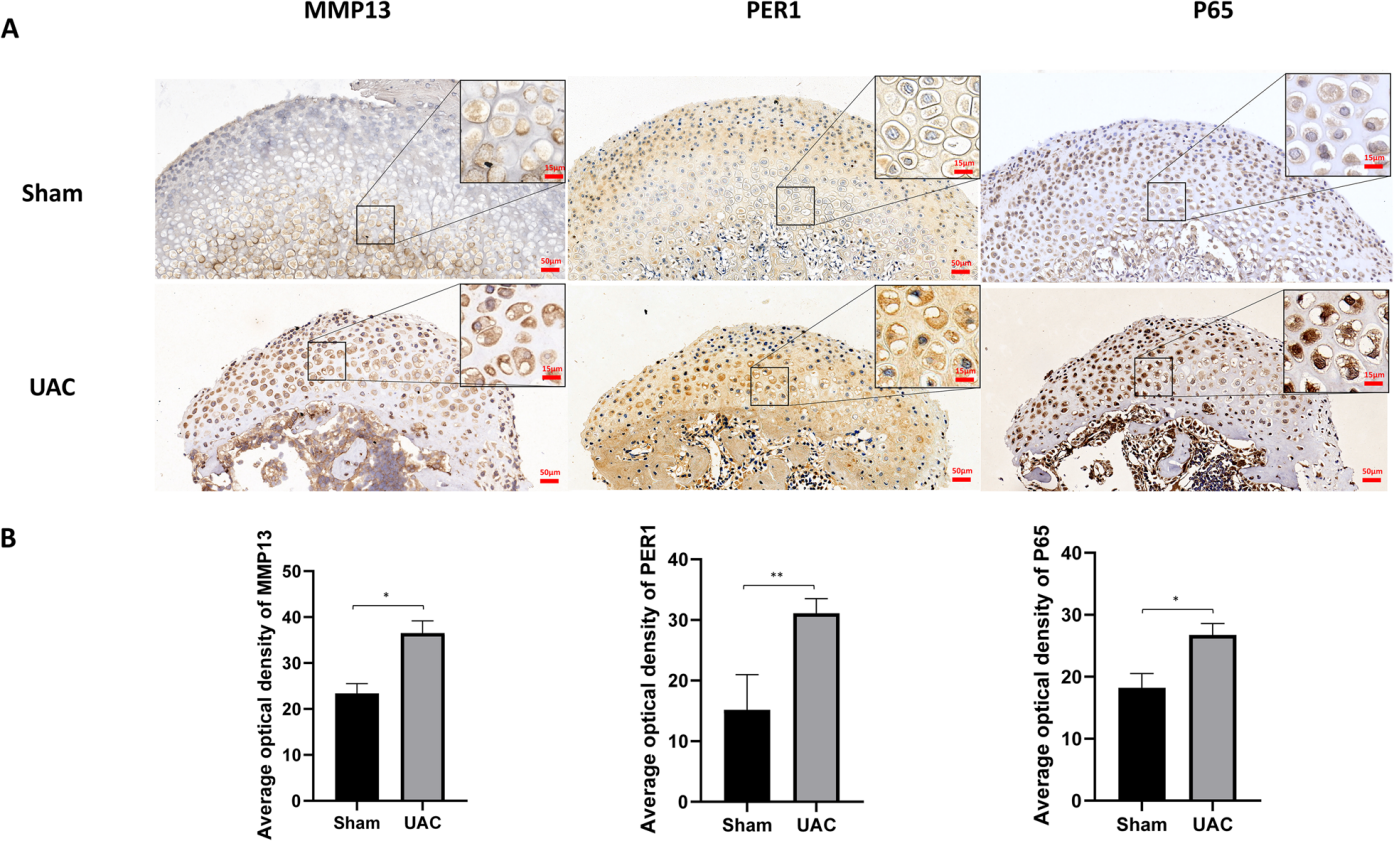
Expression of PER1, MMP13 and P65 increases in UAC-induced TMJOA rats.** A** Immunohistochemical staining of PER1, MMP13 and P65 in condyles of UAC group and Sham group. **B** The average optical density of PER1, MMP13 and P65 of temporomandibular cartilage in UAC and Sham groups (*n* = 4). Values are displayed as the mean ± SEM. *P < 0.05 and **P < 0.01

As Fig. 5B shown, compared with Sham group, the matrix degrading factor MMP13 significantly increased in mandibular condylar cartilage of UAC rats (*P* < 0.05). And clock protein PER1 also significantly increased in UAC group (*P* < 0.01). P65, the core protein of NF-κB pathway, significantly increased in cartilage of UAC group (*P* < 0.05), which is similar to the results of experiments in vitro.

## Discussion

After three-dimensional cultivation of mouse chondrocytes, a study found through gene microarray analysis that the clock genes *Per1*, *Per2* and *Clock* were expressed in chondrocytes and exhibited rhythmic expression [29]. Several studies have shown that circadian rhythm also exists in cartilage metabolism. The proliferation of chondrocytes and subchondral mineralization activities are rhythmic [16–18]. Mice with specific *Bmal1*-knockout chondrocytes show the disappearance of circadian rhythm, progressive injury of joints and decrease in bone mass of the whole body [19, 30].

Matrix metalloproteinase-13 (MMP13), mainly secreted by chondrocytes, is the most powerful COL2-degrading enzyme known in matrix metalloproteinases [31]. As mentioned above, during the decompensation phase of TMJOA, the synthesis of matrix metalloproteinases increases, a large amount of cartilage matrix is decomposed and the activity of MMP13 in chondrocytes during the progression of OA is significantly increased [32]. And MMP13 is also an important marker of terminal hypertrophy differentiation of chondrocytes [33].

This study found that *Mmp13* also showed rhythmic oscillation in mandibular condylar chondrocytes, suggesting that clock genes may affect the expression of *Mmp13*. Compared with several core clock genes, the phase of the expression curve of *Mmp13* is the closest to that of clock gene *Per1*. Therefore, we speculate that the expression of *Per1* may potentially affect the expression of *Mmp13*, and we chose *Per1* among the core clock genes for subsequent research.

Among various cytokines, IL-1β plays a crucial role in the pathogenesis of TMJOA. It can initiate an increase in the expression of matrix degrading factors including MMP3 and MMP13 in chondrocytes, making it an ideal cytokine for modeling TMJOA in vitro [34]. After stimulation with IL-1β concentration of 10 ng/mL for 24 h [24, 25], the expression of *Mmp13* significantly increased in mRNA and protein levels.

UAC rat model is a classic TMJOA modeling method, and multiple studies have confirmed that it can successfully lead to TMJOA [26, 35, 36]. In our study, the three-dimensional morphology of the condyles in the UAC group was worse than that in Sham group, with a decrease in trabecular density and bone mass of the condyles. In terms of histopathology, the modified Mankin score and thickness of cartilage of UAC group also significantly decreased compared to the Sham group.

The previous studies have shown that compared with normal individuals, the expression of clock gene *BMAL1* in the knee cartilage of OA patients is lower [19], and the expression of *PER2* increased in OA cartilage [37]. Clock genes play a crucial role in the occurrence and development of OA. *Per1* can inhibit the differentiation of pre-chondrocytes into chondrocytes by suppressing the expression of *Sox6* [38]. Silencing *Bmal1* in mouse articular chondrocytes resulted in decreased expression levels of p-SMAD2/3 and NFATC2, as well as decreased expression of chondroprotective factors and matrix-related genes such as *Sox9*, *Acan* and *Col2a1*, but increased levels of p-SMAD1/5, indicating that the clock gene *Bmal1* is involved in the maintenance and repair of cartilage. Disrupted circadian rhythms are a risk factor for OA and other joint diseases [19]. Specific knockout of *Bmal1* in cartilage promotes cartilage degradation and chondrocyte apoptosis in OA mice, inhibits chondrocyte synthetic metabolism and accelerates cartilage degeneration and osteophyte formation during OA progression [39]. Silencing *NR1D1* or *BMAL1* in human knee joint chondrocytes affects the TGFβ signaling pathway, thereby affecting cartilage homeostasis [20]. In human OA cartilage, the expression of *CRY2* increases, and it plays a positive role in maintaining the extracellular matrix homeostasis of chondrocytes [40].

We also found the expression of clock gene *Per1* increased significantly in TMJOA rat model in vitro and in vivo. After down-regulation of *Per1* in chondrocytes using lentivirus in vitro, the expression of *Mmp13* also reduced, suggesting that *Per1* may participate in the occurrence and development of TMJOA by regulating the expression of *Mmp13*. In a study, chronic sleep deprivation was found to disrupt the normal rhythm of Wistar rats, leading to TMJOA-like lesions such as osteophyte formation. Increased expression of IL-6 and p-ERK was observed in the mandibular condylar cartilage, while *Bmal1* expression was decreased. Overexpression of *Bmal1* in condylar chondrocytes suppressed the ERK pathway, indicating that *Bmal1* in chondrocytes can regulate the synthesis and secretion of IL-6 through the MAPK/ERK pathway, thereby inducing cartilage matrix degradation and participating in the pathological process of TMJOA [41]. During low-intensity pulsed ultrasound (LIPUS) treatment in rats with TMJOA, an increase in *Per2* expression was detected through RNA-seq [42]. Loss of *Bmal1* down-regulated the expression of cartilage-related genes *Prg4*, *Sox9* and *Col7a1* in bone marrow mesenchymal stem cells (BMSCs), while *Bmal1* enhanced the migration capability of BMSCs and attenuated age-related temporomandibular joint osteophyte degeneration, indicating the crucial role of circadian rhythm in maintaining condylar osteochondral homeostasis [43].

**Fig. 6:**
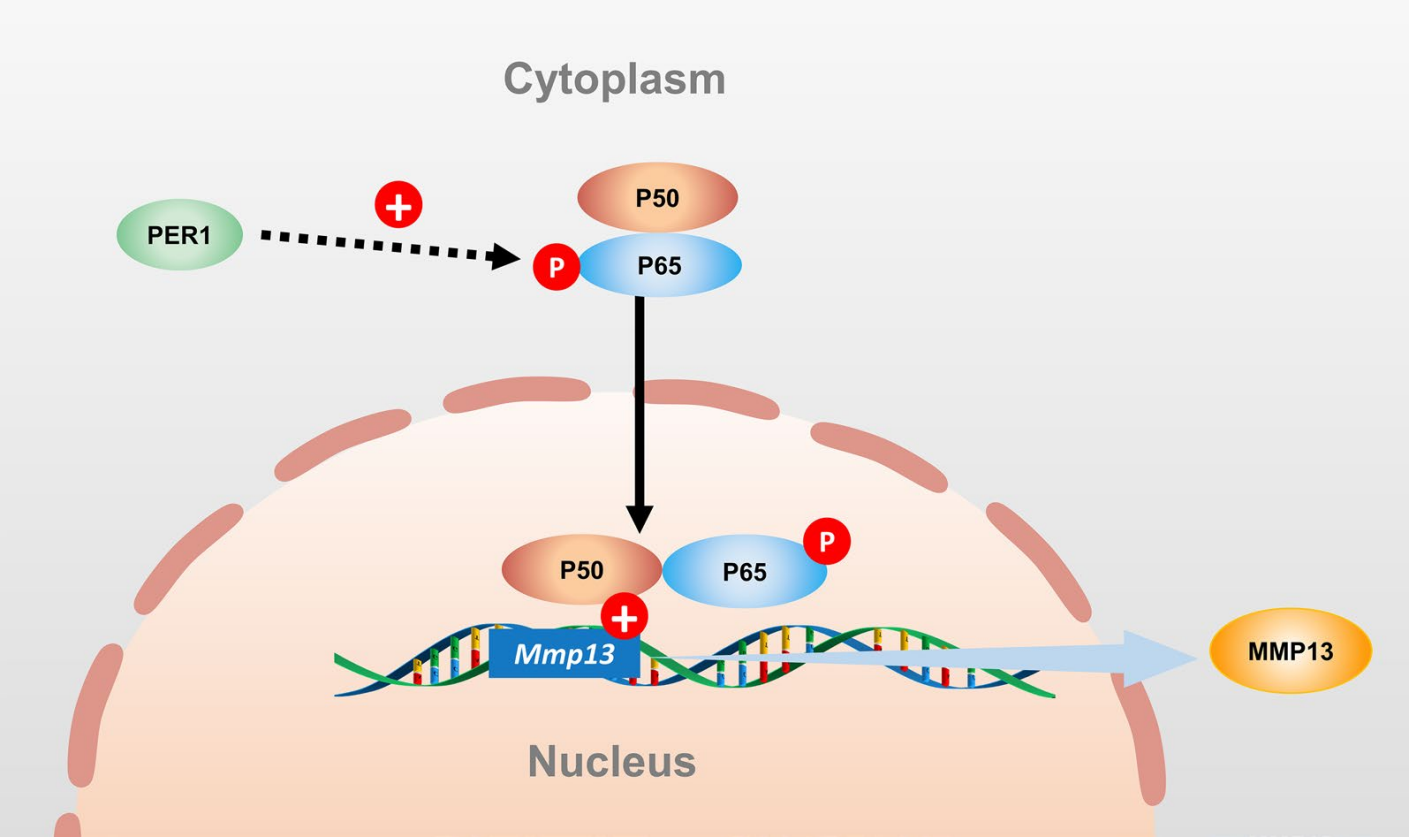
Schematic diagram of PER1-mediated increasing expression of MMP13 by modulating NF-κB pathway in rat mandibular condylar chondrocytes

The IHC results showed that both PER1 and P65 increased in the cartilage of condyles in UAC rats. And after down-regulation of *Per1* in chondrocytes in vitro, the expression of P65 and p-P65/P65 also reduced. Take these results together, as Fig. 6 shown, clock gene *Per1* can regulate the expression of MMP13 through NF-κB pathway and further to regulate the progression of TMJOA. NF-κB family plays an important role in inflammation, cell differentiation and proliferation. Human NF-κB sub-family includes five types of NF-κB transcription factors: RelA/p65, NF-κB1/p105, NF-κB2/p100, c-Rel and RelB. P65 is the core protein of NF-κB pathway. P65 and P50 can form a protein dimer. When P65 is phosphorylated, the dimer will enter the nucleus to initiate the transcription and expression of downstream genes [44, 45]. NF-κB pathway is also one of the most important signaling pathways that regulate cartilage metabolism. Activating NF-κB pathway can promote inflammation of cartilage, trigger chondrocyte apoptosis and enhance the production of matrix-degrading proteinases such as MMP1, MMP3, MMP13, ADAMTS4 and ADAMTS5, which lead to the erosion of cartilage [46–48].

PER1 is a core molecular regulator of circadian rhythm in organisms. CLOCK and BMAL1 form a positive regulatory complex that activates a series of downstream genes, including *PER1* itself. As a negative regulator of circadian rhythm, PER1, in turn, inhibits the expression of CLOCK and BMAL1, forming a feedback loop that maintains circadian clock [49]. PER1 itself is a transcriptional regulator that controls the transcription of downstream genes [50]. This study reveals that PER1 can regulate the expression of MMP13 by controlling the expression of P65 and p-P65. The mechanism involved may be PER1 regulating the transcription of a downstream molecule, which can affect the activation of the NF-κB pathway. Another possibility is that PER1 interacts with a protein in the NF-κB pathway, thereby influencing its activation. The underlying mechanisms need further in-depth research, which is also a limitation of this study.

Investigating the mechanisms by which clock genes affect TMJOA can facilitate the future development of related therapeutic or preventive drugs. Currently, there is no cure for TMJOA, only methods to alleviate symptoms. Studying the role of clock genes in TMJOA can help us develop new approaches for prevention and treatment. At the cellular level, novel drugs could directly target clock molecules in chondrocytes, influencing their molecular clock and thereby affecting chondrocyte metabolism to treat TMJOA [51]. At the systemic level, manipulating molecules that regulate peripheral circadian rhythm can regulate chondrocyte metabolism. For example, the melatonin, which regulates peripheral circadian rhythm, could be used to impact chondrocyte metabolism [52], or optimizing light–dark cycles could alleviate OA pathology caused by disruption of circadian rhythm [53]. By studying the circadian rhythm of cartilage tissue, we could optimize drug administration timing, as the target molecules for OA treatment may also exhibit circadian rhythms. Aligning the peak concentration of a drug with the peak expression of target molecules after administration could maximize the efficacy of the drug [54]. PER1 could serve as a potential target for the prevention and treatment of TMJOA or OA.

## Conclusion

Core clock genes and *Mmp13* are rhythmic expressed in rat mandibular condylar chondrocytes. PER1 can regulate the expression of MMP13 in mandibular condylar chondrocytes through NF-κB pathway. Clock gene *Per1* may be a potential target gene for TMJOA treatment.

### Supplementary Information

**Additional file 1.** Primer sequence of core clock genes and Mmp13.

## Data Availability

The data are available upon reasonable request.
