# Supplementary material for: Clock gene Per1 regulates rat temporomandibular osteoarthritis through NF-κB pathway: an in vitro and in vivo study
Source: J Orthop Surg Res. 2023 Oct 31;18:817. doi: 10.1186/s13018-023-04301-7 (PMC10619284; doi:10.1186/s13018-023-04301-7)
Supplement: Supplementary file 1 — Additional file 1. Primer sequence of core clock genes and Mmp13. [file 13018_2023_4301_MOESM1_ESM.pdf]

Tab.1 Primer sequence of core clock genes and *Mmp13*

Gene	Sequence
Mmp13	Forward：TGCATACGAGCATCCATCCC<br>Reverse：CGTGTCCTCAAAGTGAACCGC
Bmal1	Forward：GAGGCGTCGGGACAAAATGA<br>Reverse：GCTTCTGTGTATGGGTTGGTGG
Clock	Forward：ATCGGCAGCAAGAAGAACTAAG<br>Reverse：TCAGTCCAGGGTTTGATTGCT
Per1	Forward：ACATCTGAATACACTCTCCGCAAC<br>Reverse：GCAGGCGAGATGGTGTAGTAGAG
Per2	Forward：CCCAGCAAGTGATCGAGGACTA<br>Reverse：TTGACACGCTTGGACTTCAGTT
Cry1	Forward：GTCCGACGACCATGATGAGAA<br>Reverse：GCTTGCGAGCAGGGAGTTT
Cry2	Forward：ATGTGTTCCCAAGGCTTTTCAA<br>Reverse：TGTAGGTAAGGGGTGGTTTCTGC
Gapdh	Forward：CTGGAGAAACCTGCCAAGTATG<br>Reverse：GGTGGAAGAATGGGAGTTGCT
